# Study on Acoustic Emission Characteristics of Fatigue Damage of A7N01 Aluminum Alloy for High-Speed Trains

**DOI:** 10.3390/ma16124362

**Published:** 2023-06-13

**Authors:** Ronghua Zhu, Song Fang, Weibing Sun, Dazhao Chi

**Affiliations:** 1 College of Locomotive and Vehicle, Nanjing Vocational Institute of Railway Technology, Nanjing 210000, China; 2 State Key Laboratory of Advanced Welding and Joining, Harbin Institute of Technology, Harbin 150001, China

**Keywords:** aluminum alloy, acoustic emission, fatigue damage, online monitoring, AE source mechanisms

## Abstract

Online monitoring of the fatigue damage process of A7N01 aluminum alloy base metal and weld seam was conducted based on acoustic emission (AE) and digital microscopic imaging technology. The AE signals were recorded during the fatigue tests and analyzed using the AE characteristic parameter method. Fatigue fracture was observed using scanning electron microscopy (SEM) to analyze the source mechanism of AE. The AE results show that the AE count and rise time can effectively predict the initiation of fatigue microcracks in A7N01 aluminum alloy. The digital image monitoring results of a notch tip verified the prediction of fatigue microcracks using the AE characteristic parameters. In addition, the AE characteristics of the A7N01 aluminum alloy under different fatigue parameters were studied, and the relationships between the AE characteristic values of the base metal and weld seam and the crack propagation rate were calculated using the seven-point recurrence polynomial method. These provide a basis for predicting the remaining fatigue damage in the A7N01 aluminum alloy. The present work indicates that AE technology can be used to monitor the fatigue damage evolution of welded aluminum alloy structures.

## 1. Introduction

With the development of lightweight trains, aluminum alloys have been more widely used in the manufacturing of high-speed train bodies [1,2]. In the running process of high-speed trains, microcracks may initiate in aluminum alloy structures under the action of alternating loads, which can significantly reduce the train operation reliability and even cause catastrophic accidents [3]. At present, the most commonly used non-destructive testing techniques for aluminum alloy welded structures of high-speed trains include radiographic testing, ultrasonic testing, and penetration testing. Nevertheless, these methods need the trains to be immobilized for inspection, and their detection efficiency is low. Therefore, it is of urgent need to develop online diagnostic methods for aluminum alloy structures under the running conditions of high-speed trains.

Acoustic emission (AE) technology is one of the commonly used non-destructive testing methods for real-time monitoring of materials or structures. A material that bears a local load can generate instantaneous elastic waves. When an elastic wave reaches the surface of a material, it causes mechanical vibration. Sensors on a material surface can convert mechanical vibrations into electrical signals, which are then converted into AE signals after filtering and amplification [4,5,6]. Finally, the state of the material or structure can be determined based on the characteristics of the AE signals.

In practical engineering applications, the fatigue life of structures is usually predicted based on the linear elastic fracture mechanics (LEFM) method. Due to the difficulty in calculating the stress intensity factor of complex structures, the actual size and location of fatigue cracks cannot be easily determined, which introduces some trouble for engineering applications. The key to evaluating the fatigue damage state of aluminum alloys using AE technology is to determine the relationships between the AE signal characteristics and fatigue crack parameters.

Several scholars have attempted to establish relationships between AE parameters (count, amplitude, energy, rise time, etc.) and material behavior [7,8,9]. Xu et al. revealed the relationship between the fatigue crack state and AE characteristics to find suitable AE signal monitoring indices for different rail materials [10]. D’Angel and Ercolino [11] showed that AE entropy can be considered a fracture-sensitive phenomenon for the real-time assessment of metal plates under fatigue loading. Chai et al. [12] studied the stages IIa and IIb in Paris region of fatigue crack growth for base metal and weld specimens of 316LN stainless steel by using the fatigue properties and AE characteristics (AE cumulative count and cumulative energy). Aggelis et al. [13] investigated the correlation of AE parameters with damage accumulation and the fracture mode. The behavior of the RA value (ratio of rise time to amplitude of the waveforms) showed a certain shift, indicating a transition from tensile mode to shear. Moreover, Maleki et al. [14] utilized AE energy to characterize the failure of specimens and discriminated between AE signals from aluminum cracking and adhesive layer failure according to their energy content. Hou et al. [15] investigated the correlation between the characteristics of an AE signal (for example, count, energy, and amplitude) and the damage modes of a needled C/SiC composite during fatigue tests. Chai et al. [16] used AE multi-parameter analysis to study the FCG behavior of 2.25Cr1Mo0.25V steel. Song et al. [17] found that the change trend of strain was consistent with the response of AE characteristic parameters and that the fatigue compression damage caused by the deformation process of the specimen could be monitored by the change in AE characteristics. The AE parameter analysis method can provide AE source characteristics in a simple, intuitive, and quick way; however, this method only describes the local characteristics of AE signal waveforms, thus leading to the loss of a lot of information.

Waveform analysis is a method that can acquire information regarding AE sources based on time-domain waveforms of AE signals and the corresponding spectra recorded by the AE system. In waveform analysis, the waveforms of AE digital signals are recorded and analyzed; thus, this technique can be considered as an alternative to parametric analysis. In this regard, P. Antonaci et al. [18] showed that the evolution of the wavelength of the shear component of the AE signal was evaluated through the two characteristic peaks in the AE signals spectrum and the wave speed of the P or S waves. This wavelength evolution can provide information on the microcrack and macrocrack progress. Baker et al. [19] studied the initiation and propagation of cracks in carbon fiber-reinforced toughened epoxy polymer composite laminates using modal AE and waveform energies coupled with peak frequency data and correlated this to matrix crack density in the transverse direction. Furthermore, Qiu et al. [20] utilized a wavelet scalogram to analyze the irreversible characteristics of the AE process of asphalt mixtures. Kudus et al. [21] considered the b-value, improved b-value [22,23] (Ib-value), severity, and intensity analysis methods to quantify the damage level of the concrete structure under fatigue loading. These results show that transient AE analysis can be applied as a powerful and robust scheme to discriminate the damage mechanisms based on full-waveform analysis.

Herein, AE signals and crack evolution during the fatigue damage process of A7N01 aluminum alloy used in high-speed train bodies and weld seams were monitored using AE and digital microscopic image technologies. Moreover, the AE characteristics during fatigue damage of the A7N01 aluminum alloy were analyzed by the AE characteristic parameter method. Our results provide a basis for predicting the fatigue damage evolution behavior of A7N01 aluminum alloy and the corresponding weld seam.

## 2. Materials and Methods

### 2.1. Fatigue Crack Growth Test

**Figure 1:**
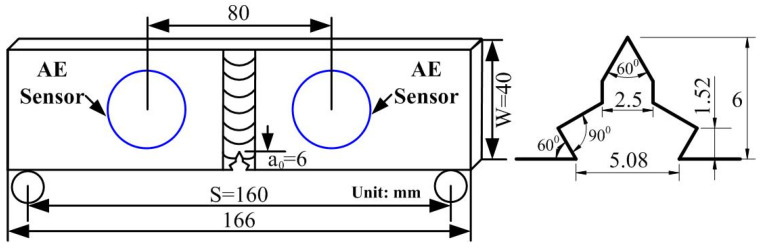
The schematic illustration of the SENB specimen and position of the AE sensor.

The testing material investigated in the present work was A7N01 aluminum alloy, which is widely used in the manufacture of high-speed train body structures. The aluminum alloy samples were welded using tungsten-electrode inert gas (TIG) welding. Single-edge notched bending (SENB) fatigue specimens of the A7N01 aluminum alloy base metal and weld (WSENB) were prepared according to the ASTM E647 standard. The geometric dimensions of the specimens and notches are illustrated in Figure 1. The notch end faces of the samples were polished with sandpaper to capture the damage images of the notch tip during the fatigue damage AE monitoring experiment.

**Figure 2:**
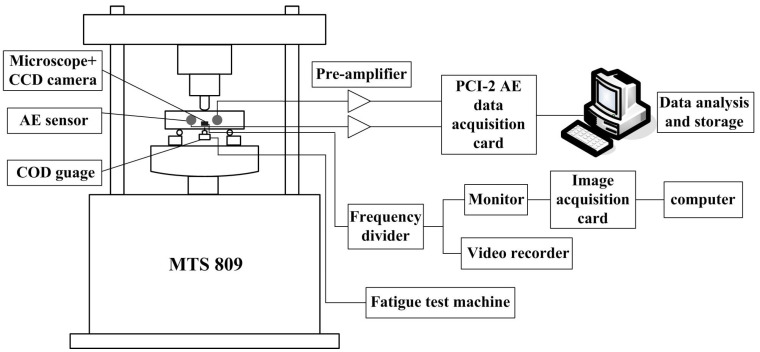
Configuration of the test set-up for fatigue damage monitoring.

Fatigue damage tests were performed on an MTS809 (MTS Systems, USA) fatigue test machine, and the test device is exhibited in Figure 2. The fatigue loading frequency of all samples was 4 Hz, and the crack length was measured using a COD gauge. The fatigue tests included whole process fatigue tests and fatigue crack propagation tests. The fatigue test parameters are listed in Table 1. In the fatigue crack propagation test, a crack of 10 mm was prefabricated using the K-decreasing method.

A microscopic image observation system was employed to conduct real-time acquisition of the damage images of the notch tip. Considering that the notch of the fatigue sample was closed once within 250 ms, one image frame was collected every 50 ms according to the Nyquist sampling theorem, in order to capture the details of fatigue crack propagation; i.e., the sampling frequency of the images was 20 Hz.

### 2.2. AE Monitoring Instrument

A PCI-2 AE system was used to monitor the AE signals during the fatigue damage process of the A7N01 aluminum alloy base metal and weld seam. Two R15I AE resonant sensors were symmetrically arranged on both sides of the prefabricated notch, with a spacing of 80 mm. Vaseline coupling was adopted between the sensors and the samples, which were fixed with adhesive tape. The signals acquired by the AE sensors were amplified by a pre-amplifier and then collected and stored on a computer.

## 3. Results and Discussion

### 3.1. Fatigue Crack Initiation and AE Characteristics

To characterize the fatigue damage degree of materials with AE signal characteristics, the AE signal characteristics during the fatigue damage process of materials need to first be investigated, and then the relationships between them and the fatigue crack parameters must be established. In engineering practice, the initiation of fatigue microcracks is often the beginning of the fatigue failure of structures; thus, the early detection of fatigue microcracks has become the focus of concern.

**Figure 3:**
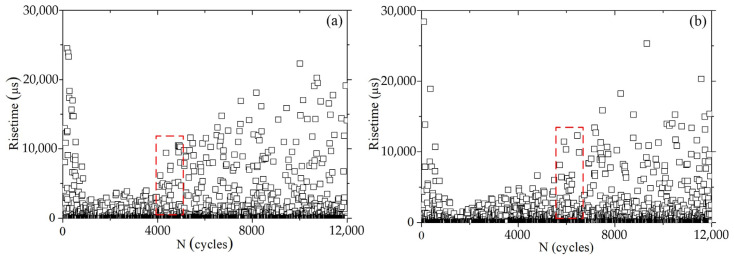
Variation diagrams of the AE rise time in the fatigue crack initiation process of A7N01 aluminum alloy: (**a**) the base metal; and (**b**) the weld.

Figure 3 presents the variation diagrams of the AE characteristic values during the fatigue crack initiation process of the A7N01 aluminum alloy base metal and weld seams. As it can be observed, the AE rise time of the base metal and weld seam increased dramatically at the initial fatigue stage, and the peak value reached 24,456 and 28,397 μs for the base metal and weld seam, respectively. This can be attributed to the friction between the testing head of the fatigue testing machine and the sample. Then, the AE rise time decreased sharply and remained at low levels of 3829 and 3489 μs for the base metal and weld seam, respectively. When the number of fatigue cycles of the base material and weld seam reached 4369 and 5908, respectively, the AE rise time increased sharply again to 9386 and 11,400 μs, as shown in the red rectangles in Figure 3a,b respectively, and then exhibited a linearly increasing trend.

**Figure 4:**
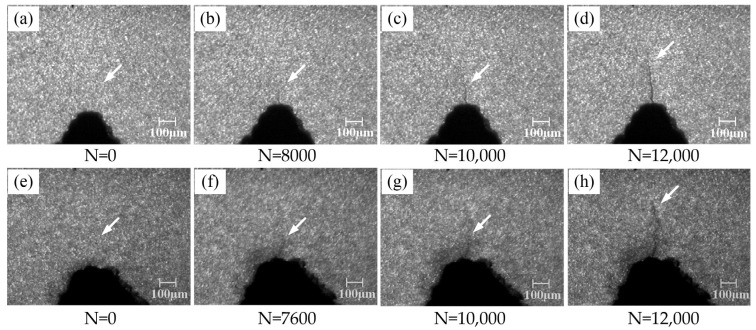
Initiation and evolution diagrams of fatigue microcracks in A7N01 aluminum alloy: (**a**–**d**) the base metal; and (**e**–**h**) the weld.

Figure 4 demonstrates the initiation and evolution of notch tip microcracks in the fatigue damage process of the A7N01 aluminum alloy base metal and weld seams. Figure 4a,b shows the initial state of the polished notch tips on the base metal and weld seam samples before fatigue loading. Under fatigue cycle stress, microcracks were found at the notch tip of the base metal after 8000 fatigue cycles (Figure 4b), and the length of the microcracks was about 87 μm. With an increase in the number of cycles, the microcrack at the notch tip of the base metal continued to propagate, and its length reached 273 μm when the number of cycles was 12,000. Compared to the evolution of the microcrack at the notch tip of the base metal, the microcrack at the notch tip of the weld seam occurred after 7600 fatigue cycles, and its length was about 127 μm. With an increase in the number of cycles, the microcrack in the weld seam continued to propagate, and its length reached 377 μm when the number of cycles was 12,000.

Table 2 lists the number of cycles and the size of fatigue microcracks in the A7N01 aluminum alloy base metal and weld seam samples when fatigue microcracks were first discovered by microscopic image monitoring and PCI-2 AE systems. As it can be observed, the minimum fatigue microcrack length detected by the micro-image monitoring system was about 87 μm. By comparing the number of cycles when fatigue microcracks were first detected by the micro-image monitoring system with that corresponding to the abrupt change in the AE characteristic value, it was found that the abrupt change in the AE characteristic value occurred earlier than the time point when the fatigue microcracks were first detected by the micro-image monitoring system. This is because the micro-image monitoring system can only detect damage on the surface of the samples, while the signals collected by the AE system are stress waves generated by internal damage to the samples. Consequently, the signals collected by the AE system can be used to evaluate the degree of damage to local regions in the samples. Overall, AE technology can be used to monitor and predict fatigue microcracks in A7N01 aluminum alloys.

### 3.2. Fatigue Crack Propagation and AE Characteristics

**Figure 5:**
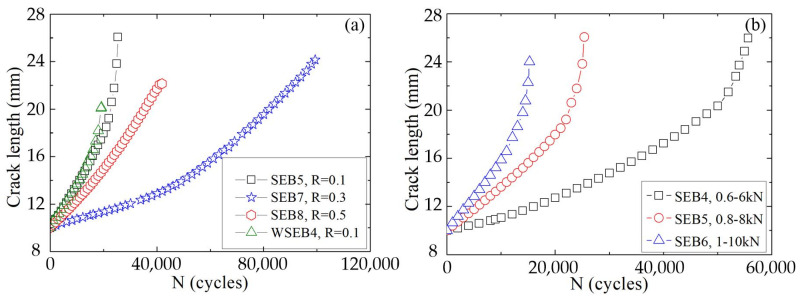
Relationships between crack length and fatigue cycle number of A7N01 aluminum alloy base metal and weld seam under different (**a**) stress ratios and (**b**) load ranges.

Figure 5 shows the variations in the crack lengths during fatigue damage of the A7N01 aluminum alloy under different stress ratios and load ranges. As can be observed, the crack propagation rate of the base metal was higher under lower stress ratios and higher peak loads, as seen in Figure 5a. In addition, under the same stress ratio, the crack propagation rate of the weld seam was higher than that of the base material, as shown in Figure 5b. This is because the impurities generated in the welding process may become the core and path of the fatigue crack nucleation and propagation, respectively.

**Figure 6:**
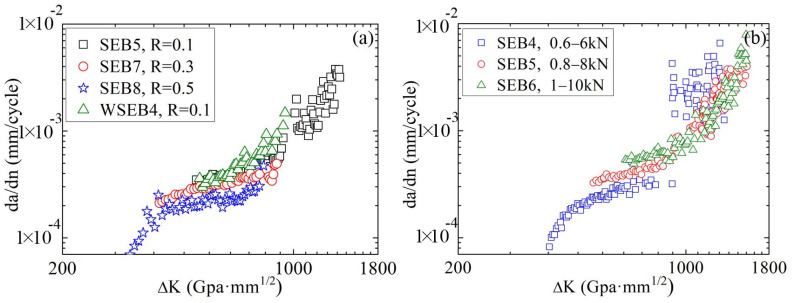
Relationships between crack propagation rate and stress intensity factor of the A7N01 aluminum alloy base metal and weld seam under different (**a**) stress ratios and (**b**) load ranges.

Figure 6 presents the relationships between the crack propagation rate and stress intensity factor of the A7N01 aluminum alloy base metal and weld seam under different stress ratios and peak loads. As it can be observed, under different stress ratios and peak loads, the fatigue crack propagation of the base metal and weld seam during the stable crack propagation stage followed the Paris rule. At the same ∆K, the crack propagation rate of the base metal increased with a decreasing stress ratio and increasing peak load.

**Figure 7:**
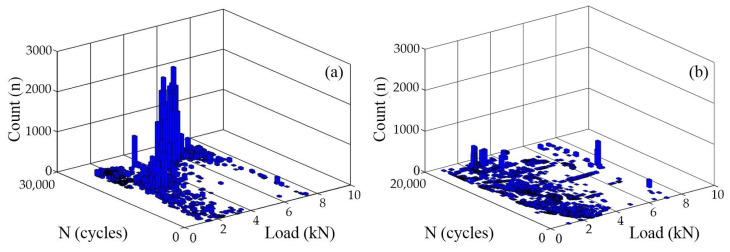
Variation in AE count with fatigue load and cycle times: (**a**) the base metal; and (**b**) the weld.

Figure 7 exhibits the three-dimensional bar charts of the AE count as a function of the fatigue load and cycle period in the fatigue damage process of the A7N01 aluminum alloy base metal and weld seam when the peak load was 8 kN and the stress ratio was 0.1. The values of the AE characteristic parameters in the three-dimensional bar charts represent the degree of activity of the AE signals under different stress states during fatigue cycles. According to Figure 5, most of the AE signals in the steady fatigue crack propagation stage were generated in the low-stress stage of the cyclic loading. This is mainly because the AE activities in the low-stress stage are mainly related to the plastic deformation and crack closure phenomenon of the crack tips. Subsequently, in the third fatigue crack propagation stage, the generation of AE signals began to shift toward the high-stress stage of cyclic loading.

**Figure 8:**
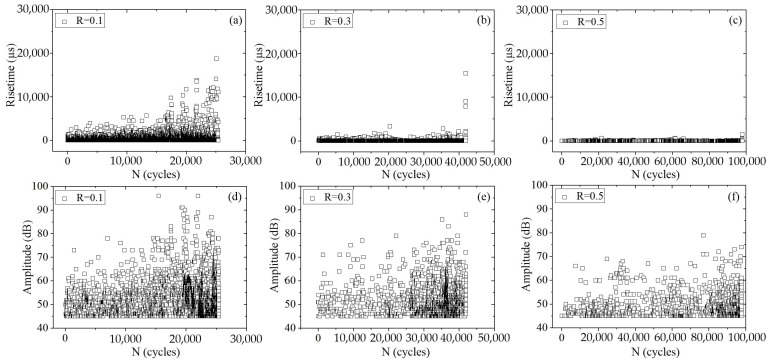
Variation in AE characteristic value for the base metal under different stress ratios: (**a**–**c**) risetime; and (**d**–**f**) amplitude.

Figure 8 presents the AE rise time and amplitude of the base material and weld seam under different fatigue stress ratios. It can be observed that, when the peak load was 8 kN and the stress ratio was 0.1, the AE rise time and amplitude during the stable crack propagation stage were in the ranges of 0–5689 μs and 45–82 dB, respectively. Whereas during the unstable crack propagation stage, they were in the ranges of 0–18,767 μs and 45–96 dB, respectively. When the stress ratio was 0.3, the AE rise time and amplitude during the stable crack propagation stage were in the ranges of 0–3381 μs and 45–79 dB, respectively, while during the unstable crack propagation stage, they were in the ranges of 0–15,482 μs and 45–88 dB, respectively. When the stress ratio was 0.5, both the AE rise time and amplitude decreased; during the stable crack propagation stage, they were in the ranges of 0–669 μs and 45–69 dB, respectively, and during the unstable crack propagation stage, they were in the ranges of 0–1490 μs and 45–79 dB, respectively. Therefore, with an increase in stress ratio, the AE rise time and amplitude tended to decrease.

### 3.3. Fatigue Life Evaluation

To predict the fatigue life of a structure based on the LEFM method, it is typically necessary to determine the initial crack, stress intensity factor, and final crack size of the structure, thus enabling the estimation of the remaining life of the fatigue crack propagation stage. Nevertheless, in practical engineering applications, it is difficult to determine the stress intensity factors of complex structures as well as the size and location of actual fatigue cracks. On account of this fact, it is not convenient to use the LEFM method for direct engineering applications.

AE technology can be applied to monitor the fatigue damage of structures. By monitoring the AE signal characteristics of actual structures in service and establishing relationships between the AE characteristic values and fatigue crack parameters, e.g., fatigue crack propagation rate, stress intensity factor amplitude, and crack length, the fatigue crack propagation rate can be determined for the evaluation of the health state of structures.

According to the linear elastic fracture theory, the growth rate of a fatigue crack can be derived from the Paris equation [24,25]:

(1) $$\frac{da}{dN}=C{\left(\Delta K\right)}^{m},$$

(2) $$\log(\frac{da}{dN})=\log C+m\log(\Delta K),$$

where *C* and *m* depend on the material characteristics and ∆*K* is the variation of the stress intensity factor that depends on the applied load *F*. Moreover, *a* and *Y* denote the crack dimension and element geometry, respectively.

(3) $$\Delta K=\frac{\Delta FS}{bW^{3/2}}*f(\alpha ),$$

(4) $$f(\alpha )=\frac{3{\left(\alpha \right)}^{1/2}[1.99-\alpha (1-\alpha )(2.15-3.93\alpha +2.7\alpha^{2})]}{2(1+2\alpha )(1-\alpha {)}^{3/2}},$$

where *α* = *a*/*W*, *b* and *W* denote the thickness and width of the test specimen, respectively. *S* is the span and ∆*F* denotes the load range.

The AE characteristics such as count and energy are also related to the crack length and the range of the stress intensity factors [24]:

(5) $$\log(\frac{d\eta }{dN})=B{\left(\Delta K\right)}^{P},$$

(6) $$\log(\frac{d\eta }{dN})=\log B+P\log(\Delta K),$$

where *η* represents the AE characteristics. Meanwhile, *B* and *P* are constants depending on the material.

The relationship between the count rate and the crack growth rate can be derived from Equations (2) and (6):

(7) $$\log(\frac{da}{dN})=\frac{m}{P}\log(\frac{d\eta }{dN})+\log C-\frac{m}{P}\log B,$$

**Figure 9:**
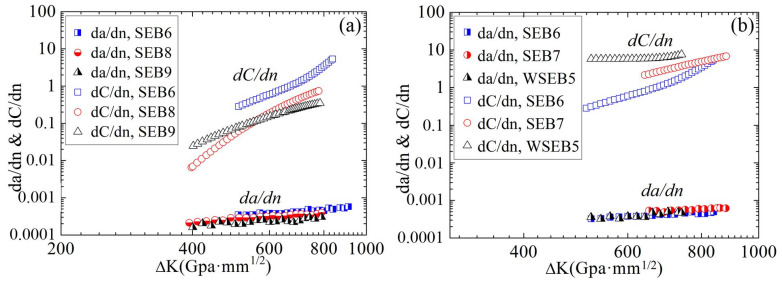
Relationships between the crack propagation rate, AE count rate, and stress intensity factor of the A7N01 aluminum alloy base metal and weld seam.

The crack propagation rate and AE count rate were calculated using the seven-point recursive polynomial method, respectively. Relationships between the crack propagation rate, AE count rate, and stress intensity factor of the A7N01 aluminum alloy base metal and weld seam were shown in Figure 9. Then the crack propagation rate, AE count rate and the stress intensity factor interval were linearly fitted. The obtained results are presented in Table 3. 

### 3.4. AE Source Mechanism of Fatigue Damage

The effects of the microstructure on the AE depend on the correlation between the AE signals, microplasticity, and microfracture events during fatigue damage. Therefore, it is important to understand the AE source mechanism in the fatigue damage process of the A7N01 aluminum alloy.

Existing studies have reported that there are three distinct stages in the evolution of AE characteristics. In the first stage, the AE comes from fatigue crack initiation. At the beginning of the fatigue test, the crack formation and the severe plastic deformation of the notch tip can produce strong AE signals, which are conducive to the rapid growth of the AE characteristic values of the samples. In addition, the stress and friction between the test head of the fatigue testing machine and the sample may generate a certain amount of noise in the AE signals.

**Figure 10:**
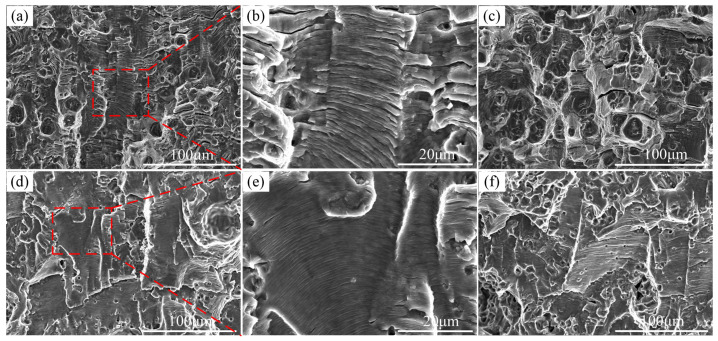
SEM observation of fracture surfaces of fatigue samples: (**a**–**c**) the base metal; and (**d**–**f**) the weld.

Stages 2 and 3 in the base metal and weld specimens are consistent with published experimental results [26]. SEM analysis of the fractured fatigue specimens of the base and weld joints was carried out to examine the AE source mechanisms in these stages. The results obtained in this regard are shown in Figure 10. Figure 10b,e are the magnified portions represented by the red rectangles in Figure 10a,d, respectively. The fracture surfaces of the base metal and weld of the 7N01 specimens in Stage 2 demonstrated that the fracture was ductile with flat facets and fatigue striations before the AE transition. Fatigue striations indicate that the crack propagation follows the plastic blunting mechanism of the tip at the crack growth stage. Therefore, AE activity depends on the slip of the plastic zone at the crack tip or a new generation of plastic zone yield edges. Figure 10 shows that as the crack propagates through the specimen, plastic deformation and stress increase, and the local stress concentration forms micro-voids and microcracks at the crack tip. The fatigue cycle aggregates micro-pores and microcracks, and dimples appear on the fatigue fracture. It should be indicated that polymeric micro-pores and microcracks have strong AE signals, thereby increasing the eigenvalues in AE Stage 3. Therefore, when the AE transition from fatigue Stage 2 to Stage 3 occurs, cracks grow by blunting and re-sharpening into the aggregates of micro-voids and microcracks.

Figure 10 indicates that fatigue striations coexisted with dimples after the AE transition, and dimples were the dominant fracture morphology in the base metal and weld specimens at the end of the fracture surface. Accordingly, it was concluded that the ductile fracture was the predominant fracture mechanism in Stage 3 after the AE transition. This issue explains why a rapid growth of AE characteristic values appears in the rapid crack expansion phase, as shown in Figure 8. The AE sources in Stages 1 and 2 were the crack initiation and deformation in the plastic zone ahead of the crack tip, respectively. Moreover, the AE source in Stage 3 mainly consisted of ligament shearing between micro-voids, microcracks, and dimples.

It is noteworthy that the fracture surfaces of the base metal and weld specimens had different morphologies. Figure 10 shows that the width and height of the fatigue striations of the weld were smaller than those of the base material in Stage 2. Meanwhile, it was found that the diameter and depth of the dimples of the welds were smaller than those of the base metal in Stage 3. This may be attributed to the smaller AE characteristic of the weld compared with that of the base metal.

**Figure 11:**
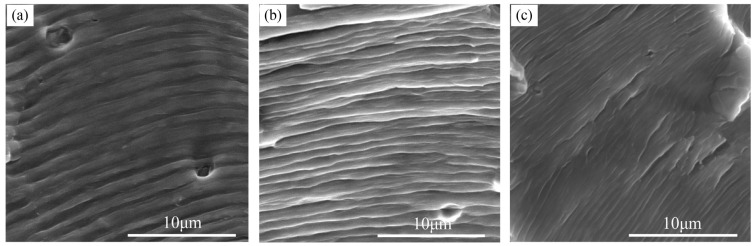
Fatigue striation spacing for the base metal under different stress ratios: (**a**) R = 0.1; (**b**) R = 0.3; and (**c**) R = 0.5.

Figure 11 shows SEM images of the fatigue fracture of the A7N01 aluminum alloy base metal under different stress ratios. It can be observed that under different fatigue stresses, the lower the stress ratio, the higher the fatigue crack propagation rate in the base material, as shown in Figure 5a. In each fatigue cycle, the large extension distance of the crack propagation manifested as an increase in the fatigue striation spacing of the fracture. This may be the main reason why the AE characteristic value of the base metal decreased with increasing stress ratio.

## 4. Conclusions

The abrupt change in the AE characteristic value appears earlier than the detection of fatigue microcracks via the micro-image monitoring system; thus, AE technology can be used to monitor and predict fatigue microcracks in the A7N01 aluminum alloy.

In the fatigue crack propagation stage, the crack propagation rate was higher, and the AE characteristic value increased severely under lower stress ratios and higher peak loads. Compared to the base material, the weld seam exhibited a faster crack propagation rate; however, the AE characteristic value was much lower. In the stable fatigue crack propagation stage, most of the AE signals were generated in the low-stress stage under cyclic loading. This is mainly because the AE activities in the low-stress stage are mainly related to plastic deformation and the crack closure phenomenon at the crack tip.

The relationships between the crack propagation rate, AE count rate, and stress intensity factor of the base metal and weld seam were calculated using the seven-point recurrence polynomial method. By appropriate conversion, the relationship between the AE count rate and crack propagation rate can be obtained, providing a basis for predicting the remaining life of fatigue damage in the A7N01 aluminum alloy.

The fatigue fracture analysis revealed that the crack tip underwent a process of repeated passivation and re-sharpening under cyclic loading. During half of the stress cycle of loading and stretching, local slip occurred at the crack tip, leading to passivation of the crack tip; during the other half of the stress cycle under opposite loading, the crack faces were pressed together, and a new surface was produced in the crack tip during loading and tension, which caused the crack tip to re-sharpen and extend forward for a certain distance. This process was continuously repeated, and the crack tip continued to propagate forward.

The sharp increase in the AE characteristic value from the second to the third stage of fatigue damage is related not only to the change in the crack propagation rate from stable to unstable propagation but also to the change in the fracture mode. Compared to the weld seam, the base material exhibited larger fatigue striation spacing and dimples. Furthermore, at lower stress ratios, the base metal presented larger fatigue striation spacing. This may be the main reason for the weak AE signals and low characteristic values of the welded sample and the strong AE signals and high characteristic values of the base material.

## Figures and Tables

**Table 1:** Fatigue test parameters.

Sample Type	Sample Number	Load Range *ΔF* (kN)	Stress Ratio	Fatigue Life *N* (Cycles)	Experimental Method
SENB specimens of the base metal	SENB1	0.8–8	0.1	69,261	Fatigue testing using specimens without pre-cracks
	SENB2	0.8–8	0.1	72,039	
	SENB3	0.8–8	0.1	74,319	
	SENB4	0.8–8	0.1	68,250	
	SENB5	0.6–6	0.1	55,676	Fatigue testing using pre-cracked specimens
	SENB6	0.8–8	0.1	25,378	
	SENB7	1–10	0.1	15,264	
	SENB8	2.4–8	0.3	41,891	
	SENB9	4–8	0.5	99,319	
SENB specimens of the weld	WSENB1	0.8–8	0.1	55,932	Fatigue testing using specimens without pre-cracks
	WSENB2	0.8–8	0.1	49,089	
	WSENB3	0.8–8	0.1	47,245	
	WSENB4	0.8–8	0.1	43,029	
	WSENB5	0.8–8	0.1	19,163	Fatigue testing using a pre-cracked specimen

**Table 2:** Statistics of microcrack size and AE characteristics in fatigue tests.

Sample Number	Minimum Crack Size Found by the MOS (μm)	Number of Cycles Corresponding to the Minimum Crack Size (N)	Number of Cycles Corresponding to the Crack Initiation for Prediction of AE Events (N)	Crack Initiation for Prediction of AE Counts (n)	Crack Initiation for Prediction of AE Duration (μs)	Fatigue Cycles (N)
SENB1	87	8000	4896	937	18,802	69,261
SENB2	121	7500	5912	1461	21,618	72,039
SENB3	138	13,000	12,173	1120	3470	74,319
SENB4	91	7100	4619	1277	22,349	68,250
WSENB1	105	7500	5532	500	4563	55,932
WSENB2	127	7600	5797	849	11,400	49,089
WSENB3	116	6700	5467	525	6641	47,245
WSENB4	93	5000	4326	403	5071	43,029

**Table 3:** The crack growth rates and AE count rate.

Sample Number	Load Range Δ*F* (kN)	Stress Ratio	Initial Crack (mm)	Fatigue Cycles	Crack Growth Rate		AE Count Rate	
					*m*	log*C*	*P*	log*B*
SENB6	0.8–8	0.1	9.990	25,378	0.79	−5.63	5.47	−15.41
SENB7	1–10	0.1	9.985	15,264	0.85	−5.69	3.40	−9.18
SENB8	2.4–8	0.3	9.995	41,891	0.81	−5.76	7.27	−20.96
SENB9	4–8	0.5	10.004	99,319	0.68	−5.52	4.29	−12.77
WSENB5	0.8–8	0.1	9.996	19,163	1.86	−8.60	1.36	−3.01

## Data Availability

The data used to support the findings of this study are available from the corresponding author upon request.
